# A systematic literature review of evidence-based clinical practice for rare diseases: what are the perceived and real barriers for improving the evidence and how can they be overcome?

**DOI:** 10.1186/s13063-017-2287-7

**Published:** 2017-11-22

**Authors:** Ana Rath, Valérie Salamon, Sandra Peixoto, Virginie Hivert, Martine Laville, Berenice Segrestin, Edmund A. M. Neugebauer, Michaela Eikermann, Vittorio Bertele, Silvio Garattini, Jørn Wetterslev, Rita Banzi, Janus C. Jakobsen, Snezana Djurisic, Christine Kubiak, Jacques Demotes-Mainard, Christian Gluud

**Affiliations:** 1 Orphanet, Institut National de la Santé et de la Recherche Médicale (INSERM), Paris, France; 2 EURORDIS – European Organisation for Rare Diseases, Paris, France; 3 Centre de Recherche en Nutrition Humaine Rhone-Alpes, Université de Lyon 1, Hospices Civils de Lyon, Groupement Hospitaler Sud, Pierre Benite, France; 4 Brandenburg Medical School, Neuruppin, and Witten/Herdecke University, Witten, Germany; 5 Institute for Research in Operative Medicine, Witten/Herdecke University, Witten and Brandenburg Medical School, Neuruppin, Germany; 6 IRCCS Istituto di Ricerche Farmacologiche Mario Negri, Milan, Italy; 7 Copenhagen Trial Unit, Centre for Clinical Intervention Research, Department 7812, Rigshospitalet, Copenhagen University Hospital, Copenhagen, Denmark; 8 Department of Cardiology, Holbæk Hospital, Holbæek, Denmark; 9 European Clinical Research Infrastructure Network (ECRIN), Paris, France

**Keywords:** Randomised clinical trials, Evidence-based clinical practice, Evidence-based medicine, Assessment, Specific barriers, Rare diseases, ECRIN, European Clinical Infrastructure Networks

## Abstract

**Background:**

Evidence-based clinical practice is challenging in all fields, but poses special barriers in the field of rare diseases. The present paper summarises the main barriers faced by clinical research in rare diseases, and highlights opportunities for improvement.

**Methods:**

Systematic literature searches without meta-analyses and internal European Clinical Research Infrastructure Network (ECRIN) communications during face-to-face meetings and telephone conferences from 2013 to 2017 within the context of the ECRIN Integrating Activity (ECRIN-IA) project.

**Results:**

Barriers specific to rare diseases comprise the difficulty to recruit participants because of rarity, scattering of patients, limited knowledge on natural history of diseases, difficulties to achieve accurate diagnosis and identify patients in health information systems, and difficulties choosing clinically relevant outcomes.

**Conclusions:**

Evidence-based clinical practice for rare diseases should start by collecting clinical data in databases and registries; defining measurable patient-centred outcomes; and selecting appropriate study designs adapted to small study populations. Rare diseases constitute one of the most paradigmatic fields in which multi-stakeholder engagement, especially from patients, is needed for success. Clinical research infrastructures and expertise networks offer opportunities for establishing evidence-based clinical practice within rare diseases.

**Electronic supplementary material:**

The online version of this article (doi:10.1186/s13063-017-2287-7) contains supplementary material, which is available to authorized users.

## Background

Clinical practice based on valid evidence is especially challenging in the field of rare diseases (RDs) [1], a group of diseases defined differently in several legislations. In Europe, diseases with prevalence equal to or lower that 5/10,000 inhabitants are considered rare [2]. In Asia, the definitions of RDs are < 1/10,000 inhabitants in Japan and Taiwan [3]. In the USA, a disease is considered rare if affecting fewer than 200,000 people, equivalent to about 6/10,000 inhabitants or less [4]. While some RDs are close to these prevalence thresholds, 10% to 20% are ultra-rare [5, 6]. The distinction between rare and ultra-rare diseases is important because of its implication in the assessment of the value of orphan medicinal products [7].

There are roughly 6000 clinically different RDs spread in all medical specialties, the largest groups being developmental defects of genetic origin, cancers, neurological diseases, systemic and rheumatologic diseases, and inborn errors of metabolism [6]. RDs are, therefore, a heterogeneous field, mostly composed of chronic and life-threatening diseases, the only feature in common being the rarity which jeopardises the performance of the research and development process when compared to common diseases [8]. This is a matter of concern, as RDs are recognised as a major health issue, and are the target of an active European Union policy [9–12]. Moreover, incentives to the drug and device industries have been put into place to boost the development of therapies for RDs in the USA in 1983 [13] and in the European Union in 2000 [2].

Recently, the International Rare Diseases Research Consortium (IRDiRC) has challenged the international research community with two major objectives: to develop the capacity to diagnose most RDs, and to establish 200 new or repurposed therapies for RDs by 2020 [14]. As of December 2015, a total of 118 orphan medicinal products have reached the market in Europe intended for about 107 diseases [15] and 432 orphan medicinal products have reached the market in the USA [16]. These results are good, but are far from meeting the needs of RD patients [17, 18]. Furthermore, the attrition of treatment options during the research and development process seems worse than with common diseases.

About 10% of the market authorisations for medicinal products for RDs are granted at a stage were the evidence is not yet firmly established through accelerated approval or conditional approval [19, 20]. Without such approvals, there is a need for the monitoring of patients treated with the new interventions for many more years. The concept of adaptive pathways has been proposed, which aims to grant marketing authorisations based on a lower weight of evidence justified by the claim that patients will have earlier access to treatment [21, 22]. Adaptive pathways are based on stepwise learning under conditions of acknowledged uncertainty, with iterative phases of data gathering and regulatory evaluations [23]. However, it has been criticised for lacking scientific support and ethical ground, and thus, for increasing uncertainty about the benefit-harm balance of new medicinal products [21, 22].

There is a need to build the evidence from basic research to bedside, through rigorous clinical research adapted to the intrinsic complexity of RDs [1]. The European Clinical Research Infrastructure Network (ECRIN) Integrating Activity (ECRIN-IA) project^1^ [24] has identified barriers for good clinical research within trials in general as well as regarding RDs, nutrition, and medical devices, and assessed how these barriers can be broken down in order to improve the production of evidence-based clinical research [25–27]. The aims of this paper are to summarise the main barriers faced by clinical research in the field of RDs and to highlight the opportunities for improvement at the European and international level (Table 1). These main barriers should be seen as additions to the barriers threatening all clinical trials, namely inadequate knowledge and understanding of clinical research and trial methodology; lack of funding; excessive monitoring; restrictive interpretation of privacy law and lack of transparency; overly complex or inadequate regulatory requirements; and inadequate clinical research infrastructures [25].

**Table 1:** Main barriers to the conduct of randomised clinical trials (RCTs) on rare diseases

Special barriers to RCTs on rare diseases	Comments
Difficult to recruit patients due to rarity	Improve patient identification through appropriate codification. Develop registries. Establish rare disease research cohorts. Improve collaboration among clinical centres. Rely on clinical research networks. And develop multinational controlled trials
Incomplete understanding of natural history to inform trial design	Develop clinical research infrastructure preparatory to clinical trials. Develop registries
Need for trial designs adapted to the small population size and clinical heterogeneity	In-depth knowledge of trial methodology, including design of *n-*of-1 trials as well as other methods designed for clinical research into rare diseases. Develop innovative, controlled study designs adapted to small population sizes and clinical heterogeneity that are acceptable by regulatory bodies. Develop clinical research infrastructure preparatory to clinical trials. Providing methodological support
Organisational challenges as a consequence from the need for multinational randomised clinical trials	Comply to Voluntary Harmonisation Procedures and to common EU regulation
Need for more sensitive outcome measures to quantify disease.	Construct rare disease-specific clinical outcome measures
Need for involvement of all the stakeholders in the study design and conduct	Involve patients as research partners to include patients’ views. Rely on European Reference Networks

Barriers as identified by European Clinical Infrastructure Network (ECRIN)

## Methods

**Fig. 1:**
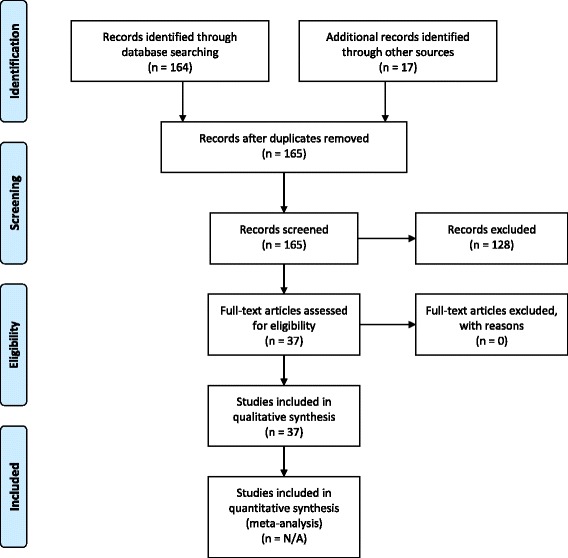
PRISMA 2009 flow diagram. PRISMA flow diagram depicting the selection process of relevant academic literature

The present paper is based on personal ECRIN communications during four face-to-face meetings and six telephone conferences from 2013 to 2017, and systematic literature searches in May 2016 for appropriate articles using the following databases: The Cochrane Library (Wiley) (Issue 5 of 12, 2016) (including the Cochrane Database of Systematic Reviews (CDSR)), CENTRAL, National Health Service Economic Evaluation Database (NHSEED), and Database of Abstracts of Reviews of Effects (DARE, U.S. Library of Medicine); MEDLINE (Ovid SP) (1946 to May 2016); EMBASE (Ovid SP) (1974 to May 2016); and Science Citation Index Expanded (1900 to May 2016), using different terms covering barriers, evidence-based medicine, and RDs. No meta-analyses were performed. The exact search strategy is provided in Additional file 1. A PRISMA flow diagram depicting the selection process and a PRISMA Checklist are provided in Fig. 1 and Additional file 2. Articles obtained from the systematic literature search, which were relevant to the field of RDs, were included in Additional file 3. Articles were selected and referenced in the review if they if they contributed to the discussion and conclusions drawn by the ECRIN expert panel, and included valid considerations on how barriers to the conduct of randomised clinical trials (RCTs) on RDs could affect their number, feasibility, and quality. The results are described narratively, which is a limitation of the data collected.

## Results and discussion

### Search results

The systematic searches identified a total of 148 references. The screening process narrowed the academic literature search down to 37 relevant references listed in Additional file 3. Characteristics of included references: overviews and narrative reviews.

### Main barriers related to clinical trials for rare diseases

#### Recruitment issues: a direct consequence from rarity

Clinical trials on RDs are characterised by an intrinsic difficulty to identify patients. This problem resides in the difficulty to diagnose RDs, to record diseases, and to trace RD patients [28]. This is due in part to the scarce knowledge about these diseases, and to the fact that far from all countries have efficient processes for referral [29], resulting in significant delays in diagnosis. RD patients often remain undiagnosed even in the best conditions of expertise due to lack of knowledge about natural history or clinical signs and symptoms. However, the most recent technological developments such as lower-cost, next-generation gene sequencing is increasing the diagnostic capacity for monogenic diseases, thereby contributing to increased knowledge of potentially actionable ethiopathogenic mechanisms [30].

In all cases, RDs are poorly represented in medical nomenclatures used in health information systems [28], making it difficult to identify participants for clinical research from medical records. Most countries use the *International Classification of Diseases* (ICD-10) to record patients, where around 500 RDs have a specific code [31]. In countries using Systematized Nomenclature of Medicine (SNOMED), the situation is not much better because only around 40% of RDs are listed here (Ana Rath, personal communication on the Orphanet-SNOMED CT mapping exercise, August 2015).

Another source for identification of RD patients is disease-specific patient registries. There are 690 such registries in Europe, covering 984 RDs [32]. Most are national (482 registries), or regional (75 registries), with some being European (59 registries) or international (74 registries). However, quality, scope, and capacity of many registries are limited [28].

The geographical dispersion of patients requires multicentric, multinational collaboration, introducing additional regulatory and funding barriers. For severe RDs, travel to research centres may pose an insurmountable barrier to research participation. Solutions include the leveraging of technology to monitor patients remotely, and setting up community centres to better deliver these trials to patients who otherwise would be unable to access them [33]. Effective recruitment is also supported through partnership with patient organisations when they exist, but also with patient registries and centres of expertise.

These barriers hamper recruitment into clinical trials. In Europe, a voluntary policy has been undertaken in order to improve diagnostic rates, i.e. by enhancing the expertise of specialised centres, and to establish European Reference Networks (ERNs) expected to spread expertise and share best practice. ERNs are expected to catalyse the international cooperation and patient engagement needed for clinical research. In parallel, in order to increase the visibility of RD patients in health information systems, a specific standard nomenclature for RDs – the Orphanet nomenclature [34] – is promoted in the European member states [35]. The implementation of the Orphanet nomenclature of RDs (the Orphacode) which is linked to other nomenclatures and resources used both in the clinical setting (ICD-10, SNOMED Clinical Terms) and in the research setting (Online Mendelian Inheritance in Man (OMIM); Human Gene Nomenclature Committee (HGNC); Universal Protein Resource (UniProtKB); among others [31]) will make it possible to more easily identify patients from health records for clinical research. The Orphanet nomenclature should also enable data exploitation with the aim of improving knowledge of the natural history of RDs.

#### Limited knowledge on natural history of rare diseases

The natural history of most RDs is often difficult to document, yet it is a necessary step to inform to the trial design for the disease. Few relevant epidemiological studies are published due to the difficulty of identifying and documenting patients widely spread geographically, not always diagnosed properly, and rarely followed-up by academic centres in a systematic way. Most attempts to collect good quality data are supported by short-term grants, which do not allow continuity in the effort. The high cost of high-quality natural history studies has been a significant obstacle to their conduct. This is well identified as a barrier requiring solutions and has been the target of recommendations of the EU Committee of Experts on Rare Diseases (Commission Expert Groups on Rare Diseases (CEGRD), formerly EUCERD) [36] and of the International Rare Diseases Research Consortium [37]. The lack of natural history information provides little insight into how to choose outcomes or how to design and power a clinical trial. When disease-specific registries meet quality standards, their relevance for contributing to high-quality clinical trials is demonstrated [38]. Structure and design of natural history studies are pivotal to capture clinical information efficiently in order to be used in safety and efficacy determination. Knowledge of natural history is one of the first crucial steps for building evidence as it allows for a better choice of clinically relevant outcomes as well as of the duration needed to monitor for them to occur [33]. There is a need to capture clinical information more cost-efficiently and to help inform the optimal approach to treatment development. Data collection in the framework of European Reference Networks should be encouraged and facilitated by common interoperability standards and tools to address this issue.

#### Need for trial designs adapted to the small population size and clinical heterogeneity

RCTs are the goal standard for producing evidence on the efficacy of an intervention because they have a strong internal validity by minimising bias and confounder factors [1, 39]. Systematic reviews of RCTs provide the highest level of evidence assessing the benefits and harms of interventions [1]. However, randomisation can prove to be difficult with RDs, mostly because of the small size of the patient population.

The European Medicines Agency (EMA), in a guideline on trials in RD populations, stated that there are no methods relevant for small trials that are not also applicable to large studies [40]. The problem for trials in RD populations is that the reverse would lead to requests for sample sizes that are not practicable, or simply impossible to reach.

The traditional RCT designs are difficult to conduct in small populations because it is very difficult to create homogeneous groups and to adequately assess changes between variable groups. Alternative methods have been proposed and could be applicable under certain conditions. We will briefly discuss some of them here. For in-depth analyses and comparisons between some of these different trial designs, see references [39–57].

The traditional fixed error rates (alpha = 0.05; beta = 0.20) cannot capture all desirable inferences in different clinical research settings. Therefore, Ioannidis and colleagues have developed models that optimise the selection of type I and type II errors according to available sample size and a plausible intervention effect [50].

Controlled rigorous designs that allow within-patient comparisons and treat all participants would assess therapies more accurately if feasible. Such study designs comprise *n*-of-1 designs and crossover trials. Both assess efficacy of a treatment based on short-term outcomes and mitigate the effects of clinical heterogeneity in a patient population [39, 40].

Pragmatic RCTs could represent an alternative to early phase RCTs while keeping most of their methodological advantages. These pragmatic trials are intended to inform decisions in common practice, so eligibility criteria are more inclusive, comparisons are done against standards of care instead of placebo, and follow-up tends to evaluate longer-term effects than early phase RCTs [39].

Vickers and Scardino and Potter et al. argue for a wide adoption of what they call ‘clinically integrated’ or ‘hybrid design’ RCTs [39, 51]. These trials incorporate aspects of observational and interventional trials (for instance, cohort multiple randomised controlled trials – cmRCTs), thus allowing for a more efficient knowledge transfer into real-world clinical practice. The designs promote longitudinal observational data collection (registries and cohorts). Such investment would be, in our view, the most efficient use of resources in the long term, as it allows for a better understanding of diseases and for the assessment of different interventions over time in a controlled way while knowledge progresses.

Other study designs more focused on proving the efficacy of interventions, more often drugs that are expected to transform the disease course, include adaptive designs. Response-adaptive methods change allocation ratios depending on which treatment appears to be best. Adaptive methods are defined by the EMA as a ‘statistical methodology (that) allows the modification of a design element (e.g. sample size, randomisation ratio, number of treatment arms) at an interim analysis with full control of type I error’ [52]. Adaptive trials are complex and need even stronger measures to prevent biasing adaptive decisions in the course of the trial [53]. Mauer and EORTC collaborators, for instance, point out the fact that regulatory and financial management need to be adaptive as well, so such trials increase the organisational and economical burdens [50]. Adaptive methods rely on real-time data, which may be easier in RD trials because recruitment tends to be slower. Some adaptive designs are now used for rare cancers [53]. Other sequential adaptive methods are proposed for testing different therapeutic possibilities in a small population [54, 55]. Regulators accept or recommend some of these designs [56]. For an in extenso review of the different designs available and their acceptance by regulatory bodies (FDA, EMA) please see Billingham et al. [57].

The RD field needs the development of cost-efficient, novel, rigorous controlled trial designs and relevant analyses that are effective in studying efficacy in heterogeneous, small populations. Recently, the European Commission funded three projects in this area [58–60]. In addition, the IRDiRC consortium has established a task force to address the question and produce recommendations [61].

As for any other disease, the laws of probability and statistics apply to RDs. Therefore, valid evidence on interventions requires valid clinical research in the form of large, well-conducted RCTs [1, 7, 39, 45].

#### Organisational challenges: a consequence from the need for multinational randomised clinical trials

Patients with most RDs are not so few as to prevent conducting large RCTs. In the EU, a prevalence of 1/100,000 with a RD (i.e. well below the threshold of 5/10,000) results in an availability of 5000 potential trial participants [62]. It requires, however, multinational cooperation, which introduces a new line of barriers in the form of comprehensive organisational, regulatory, and economical requirements. The identification of partners having both the expertise and the capacity to conduct international RCTs, the organisation of the collaboration, and also of the monitoring and follow-up are challenging. The collection and maintenance of high-quality data among all parties involved is a major issue, and specific measures should be put in place to ensure the best, easy-to-use quality. These challenges are greater in RDs, as they often need a multi-disciplinary management team as well as professionals from diverse hospital departments, which makes monitoring and organisation more complex.

Different legal frameworks in different countries contribute to the regulatory barriers of conducting multicentre international RCTs. Heterogeneity can involve all the following: ethics committee submission, patient information and consent, insurance acquisition, activation of the clinical centre, data protection rules, and investigator reimbursement. The need for harmonised procedures has been addressed by setting the Voluntary Harmonisation Procedure (VHP) in the European Union in order to organise the assessment of multinational RCTs, which is the responsibility of the Clinical Trials Facilitation Group of the Heads of Medicines Agencies (HMA) [63]. From 2009 to 2015, 22% of European clinical trials underwent that procedure, the numbers having increased impressively over time [64]. When the European clinical trials regulation is implemented in 2018 [65], the need for VHP is expected to decrease or completely disappear.

#### Need for more sensitive outcomes to quantify clinical benefit

Maybe more than in other fields, RDs are often characterised by important clinical variability, including age of onset, severity, speed of evolution, responsiveness to treatment, global impact in health status, and functional consequences. This situation leads to a very large range at baseline for many measures of efficacy, making it hard to detect important changes of an intervention. In fact, traditional RCTs assess average treatment impact in selected patients, and thus do not accommodate clinical heterogeneity very well [39]. Researchers often use surrogate outcomes to measure the effects of an intervention [66]. Such surrogates must be correlated to a clinically meaningful outcome. However, correlation alone does not make a surrogate valid [66]. Intensive analyses linking the intervention effect on the surrogate to patient-centred outcomes are needed [66–69]. Biomarker development is one source for potential surrogate outcomes. FDA and EMA orphan drug regulations contemplate approval of drugs for which the benefits for patients with unmet medical needs are based on reasonable evidence, often based on surrogate outcomes, that should demonstrate their clinical benefit during post-marketed studies [29]. Such ‘adaptive’ pathways and procedures seem to have their special problems making them less attractive or outright dangerous to patients [21, 70]. Drug or medical device companies could base their marketing authorisation applications on uncontrolled or controlled observational studies rather than pivotal RCTs. Such applications could lead to marketing approval of interventions that are without effect or that are even harmful. Such interventions are difficult or impossible to remove from the market.

However, both patients and decision-makers will seek more patient-centred, clinically relevant benefits [39]. These patient-centred outcomes can be reported by clinicians (clinician-reported outcome measures) or other observers (observer-reported outcome measures), or by the patients themselves (patient-reported outcome measures, PROM) [71].

On the other hand, the frequent complexity of disease manifestations in multiple body systems may require more than one clinical outcome for one domain to adequately assess a clinically effective treatment. That puts extra burden on the statistical assessment of outcomes [72]. As single clinical outcomes may not adequately cover the multiple expression of a disease, novel approaches to combine independent clinical outcomes in multi-domain analyses could potentially help assessing the clinical efficacy of an intervention. However, such analyses are statistically complex, the weight of each clinical variable could not be adequately measured, and results could be difficult to compare from trial to trial [73]. Nevertheless, the development of multiple domain outcome strategies in smaller populations offers important advantages over single primary outcomes [74]. Well-chosen and designed multiple domain outcomes would capture broader therapeutic data, provide greater insight into overall treatment effects, and allow successful small trials with compelling new treatments when the benefit might be varying between individual patients.

The development of agreed standardised sets of outcomes, the core outcome sets (COS) would result in better comparability between clinical studies, by defining the minimum outcomes that should be assessed when evaluating a new intervention. Initiatives like COMET develop both COS and a consensus core outcome sets database in which several RDs are represented [75]. A task force on patient-centred outcome measures have been set up by the IRDiRC, and a first overview and recommendations document has been open to public consultation [67]. The landscape of initiatives on the matter, including those concerning RDs, is depicted [71]. The International Society for Pharmacoeconomics and Outcomes Research (ISPOR)[76] has set up a task force on Rare Disease Trials Clinical Outcome Assessment (COA) measurement [77]. This task force aims to provide recommendations on the development of patient-centred outcome measures (PCOMs) in conformity to the regulatory guidance for the evaluation and proof of treatment benefits for medicinal products approval. The recent recommendations of the ISPOR Pediatric PRO Task Force [78] provide good research practices in developing and implementing paediatric patient-reported outcomes instruments and, therefore, is of interest for RDs, because most of them are paediatric.

Understanding the clinical meaningfulness of clinical changes in a patient is difficult without significant prior clinical experience. A systematic approach using natural history and comparable disease information has to be developed. The construction of the future evidence starts by the collection of natural history data in a systematic way (registries, cohorts) and by the capture of data from clinical records in a structured way. Patient engagement should be sought and encouraged from this early stage.

#### Need for involvement of all the stakeholders in the study design and conduct

The design and specific methodological aspects of a clinical trial need to be carefully discussed with all relevant partners. As stated in Potter et al. [39] ‘*ideally, evaluative research should incorporate outcomes that are of greatest importance with respect to treatment goals, based on a consensus among patients, clinical providers, researchers, and policy decision-makers*’. The final goal is to translate knowledge into clinical practice, based on the best evidence. Doing this requires effective interaction among stakeholders from the earliest phase, i.e. data collection to increase the knowledge of each RD. Thus, databases and registries should incorporate patients’ views. Some experiences exist already in which patients contribute directly to data collection [79].

Usually, a significant proportion of those with an RD must be enrolled in trials to reach the required sample size. The relationship between the clinician and the patient needs to be based on mutual trust for the patients to agree to take part and once in the trial, to stay in and provide outcome data. These data must answer a question that is important for patients, clinicians, and policy-makers, and data must be collected in such a way that taking part in the trial leaves a participant willing to take part in more.

A trial involving a RD population must, in short, be compellingly efficient and involve all stakeholders, especially patients, in its design. The regulators as well should be included in the discussion about the most appropriate design for a specific trial, as early as possible in the research process. Protocol assistance and scientific advice from regulatory bodies have been shown to play a key role in guiding the conduct of studies to address the benefit/risk analysis for marketing authorisation and approval [80]. However, the scientific advice provided by regulatory authorities poses serious concerns about conflict of interests as it is delivered by the very same agency that will grant the marketing approval at a later time point.

Centres with expertise in RDs should play a major role in fostering clinical research networks and infrastructures and in disseminating and sharing study outcomes. Training of investigators and patients’ representatives will ensure a better understanding of regulatory, methodological, and ethical requirements. The development of European reference networks in the coming months offers an opportunity to put this statement into practice. In addition, support from organisations, such as ECRIN, can greatly enhance the organisation and management of multinational clinical trials on RDs. In effect, ECRIN brings together national networks of clinical trial units across Europe, making it possible to accelerate patient recruitment and trial implementation, while ensuring the appropriate management services for smooth trial conduct. By using ECRIN (or a similar infrastructure), there is an opportunity to develop common and harmonised practices for the submission, monitoring and reporting of multicentre and multinational RD clinical trials.

## Conclusions

The main barriers described above for conducting RCTs in RD patients should be seen as additions to the barriers facing all clinical trials: inadequate knowledge and understanding of clinical research and trial methodology; lack of funding; excessive monitoring; restrictive interpretation of privacy law and lack of transparency; overly complex or inadequate regulatory requirements; and inadequate clinical research infrastructures [25].

The area of RDs is in particular need of a concerted approach of all interested parties as the challenges due to rarity are especially complex. There is a need for solid evidence before offering innovative treatments to patients [73]. The difficulties can only be overcome if a multi-stakeholder dialogue is going on, which is the recommendation of the EUCERD [81] and ICORD [82]. This multi-stakeholder approach is needed from the earliest stages of the construction of evidence, before any clinical research is commenced. Data production and collection in a way they can be shared, exploited, and re-used is a key issue to increase the knowledge of the natural history of each RD, thus identifying clinically relevant indicators for patient-centred care based on evidence [83]. Identification of optimal future outcomes is mandatory when designing future trials. Simulations may help to decide on the most appropriate study design [84]. Patient registries or systematic collections should take into consideration the views of patients. The establishment of multicentre databases/registries in a structured way, taking into account the requirements of high-quality clinical research and of regulatory exigencies, is mandatory to achieve good-quality clinical research [85]. The future European reference networks could provide a timely opportunity to work this way with the aim to conduct clinical ‘research done differently’ [86], meaning clinical effectiveness research (CER) with a clear focus on including stakeholders in the planning activities. They should also be the place for implementing educational training on clinical research for clinicians which is an already identified limitation in the conduct of multicentric RCTs [87].

However, clinical research for RDs poses specific problems that need to be addressed, and they have been roughly summarised in this paper. Multinational RCTs are needed in order to increase the size of the population studied despite the fact that this may create organisational, monitoring, and regulatory burdens. Tools and support provided by ECRIN are aimed to help overcome these barriers. Several multinational RCTs on RDs are already being conducted with the support of ECRIN [24].

If the data originate from small populations, there are special problems when two or more RCTs should be meta-analysed [88]. In these situations, special analytic methods need to be considered [88]. In these situations, using frequentist methods, it is also important to assess data with Trial Sequential Analysis to control random type I and type II errors due to sparse data and repetitive testing [1, 89, 90]. For a given required information size, a corresponding number of required trials exist. By increasing the number of trials, one can increase the power of a random-effects model meta-analysis [91].

Addressing the specific problems posed by RD clinical research is of paramount importance to provide best practice medical management to these patients. Improvements of the methodologies used for establishing evidence-based clinical practice within RD will also benefit clinical research on ‘personalised medicine’ for more common diseases. We need to make published clinical research become more valid, and to get all clinical research published, including research with negative results [1, 83, 92–98].

## Additional files

Additional file 1: Academic literature search strategy. Exact search strategy applied for analyses. (DOCX 13 kb)

Additional file 2: PRISMA 2009 Checklist. (DOC 63 kb)

Additional file 3: Relevant references from academic literature search. Results listed from literature search in the form of relevant publications. (DOCX 18 kb)

## Abbreviations

CDSR: Cochrane Database of Systematic Reviews
CEGRD: Commission Expert Groups on Rare Diseases
CER: Clinical effectiveness research
cmRCT: Cohort multiple randomised controlled trial
COA: Clinical outcome assessment
COMET: Core Outcome Measures in Effectiveness Trials
COS: Core outcome sets
DARE: Database of Abstracts of Reviews of Effects
ECRIN: European Clinical Infrastructure Network
EMA: European Medicines Agency
EORTC: European Organisation for Research and Treatment of Cancer
EUCERD: European Union Committee of Experts on Rare Diseases
FDA: Food and Drug Administration
HGNC: Human Gene Nomenclature Committee
HMA: Head of Medicines Agencies
ICD: *International Classification of Diseases*
ICORD: International Conference on Rare Diseases and Orphan Drugs
IRDiRC: International Rare Diseases Research Consortium
ISPOR: International Society for Pharmacoeconomics and Outcomes Research
NHSEED: National Health Service Economic Evaluation Database
OMIM: Online Mendelian Inheritance in Man
PCOM: Patient-centred outcome measures
PRO: Patient-reported outcomes
PROM: Patient-reported outcome measures
RCT: Randomised clinical trial
RD: Rare diseases
SNOMED: Systematized Nomenclature of Medicine
UniProtKB: Universal Protein Resource
VHP: Voluntary Harmonisation Procedure

## Footnotes

1 Funded by the European Union Framework Programme 7 (EU FP7; grant agreement no. 284395), ECRIN-IA involved 23 countries and brought together diverse stakeholders to overcome barriers to clinical research in three particularly difficult areas (rare diseases, medical devices, and nutrition). Specifically, the project aimed to develop tools, services, and infrastructure to facilitate multinational clinical research in Europe, and to support the development of pan-European disease networks to drive clinical projects. This in turn was intended to improve Europe’s attractiveness to industry, boost its scientific competitiveness, and result in better healthcare for European citizens. Originally planned for 4 years (2012 to 2015), the clinical trials work package was extended until 2017.
