# Supplementary material for: A systematic literature review of evidence-based clinical practice for rare diseases: what are the perceived and real barriers for improving the evidence and how can they be overcome?
Source: Trials. 2017 Nov 22;18:556. doi: 10.1186/s13063-017-2287-7 (PMC5700662; doi:10.1186/s13063-017-2287-7)
Supplement: Supplementary file 3 — Relevant references from academic literature search. Results listed from literature search in the form of relevant publications. (DOCX 18 kb) [file 13063_2017_2287_MOESM3_ESM.docx]

Additional file 3. Relevant references from literature search (n=37)

- Augustine EF, Adams HR, Mink JW. Clinical Trials in Rare Disease: Challenges and Opportunities. Journal of Child Neurology. 2013;28(9):1142-50.
- Auld J, Rose M, Seyedsadjadi R. Registry of Outcome Measures (ROM); tools supporting review and selection of outcome measures (OMs) for studies and trials. Orphanet Journal of Rare Diseases. 2010: Conference: 5th European Conference on Rare Diseases, ECRD 2010 Krakow Poland.
- Bajard A, Chabaud S, Cornu C, Castellan AC, Malik S, Kurbatova P, et al. An in silico approach helped to identify the best experimental design, population, and outcome for future randomized clinical trials. Journal of Clinical Epidemiology. 2016;69:125-36. Epub 2015/07/19.
- Billingham L, Malottki K, Steven N. Research methods to change clinical practice for patients with rare cancers. The Lancet Oncology. 2016;17(2):e70-80. Epub 2016/02/13.
- Bolignano D, Pisano A. Good-quality research in rare diseases: trials and tribulations. Pediatr Nephrol. 2016. Epub 2016/01/29.
- Bollyky TJ, Cockburn IM, Berndt E. Bridging the gap: improving clinical development and the regulatory pathways for health products for neglected diseases. Clinical Trials (London, England). 2010;7(6):719-34.
- Buckley BM. Clinical trials of orphan medicines. Lancet. 2008;371(9629):2051-5. Epub 2008/06/17.

Cornu C, Kassai B, Fisch R, Chiron C, Alberti C, Guerrini R, et al. Experimental designs for small randomised clinical trials: an algorithm for choice. Orphanet Journal of Rare Diseases 2013;8:48.

- Davis C, Lexchin J, Jefferson T, Gotzsche P, McKee M. "Adaptive pathways" to drug authorisation: adapting to industry? BMJ. 2016;354:i4437. Epub 2016/08/18.

Demotes-Mainard J. Clinical research networks as a support to independent clinical trials. Clinical Therapeutics. 2015;Conference: 12th Congress of the European Association for Clinical Pharmacology and Therapeutics, EACPT 2015 Madrid Spain.

- Ensor H, Lee RJ, Sudlow C, Weir CJ. Statistical approaches for evaluating surrogate outcomes in clinical trials: A systematic review. Journal of Biopharmaceutical Statistics. 2015:1-21. Epub 2015/09/24.
- Friede T, Rover C, Wandel S, Neuenschwander B. Meta-analysis of few small studies in orphan diseases. Research Synthesis Methods. 2016. Epub 2016/07/01.
- Haffner ME. Orphan Drug Development - International Program and Study Design Issues. Drug Information Journal. 1998;32(1):93-9.
- Hall AK, Carlson MR. The current status of orphan drug development in Europe and the US. Intractable & Rare Diseases Research. 2014;3(1):1-7. Epub 2014/10/25.
- Hlavin G, Koenig F, Male C, Posch M, Bauer P. Evidence, eminence and extrapolation. Statistics in medicine. 2016;35(13):2117-32. Epub 2016/01/13.
- Imberger G, Thorlund K, Gluud C, Wetterslev J. False-positive findings in Cochrane meta-analyses with and without application of trial sequential analysis: an empirical review. BMJ Open. 2016;6(8):e011890. Epub 2016/08/16.
- Ioannidis JP, Fanelli D, Dunne DD, Goodman SN. Meta-research: Evaluation and Improvement of Research Methods and Practices. PLoS Biology. 2015;13(10):e1002264. Epub 2015/10/03.
- Joppi R, Gerardi C, Bertele V, Garattini S. Letting post-marketing bridge the evidence gap: the case of orphan drugs. BMJ. 2016;353:i2978. Epub 2016/06/24.
- Keat N, Law K, McConnell A, Seymour M, Welch J, Trimble T, et al. International Rare Cancers Initiative (IRCI). Ecancermedicalscience. 2013;7:ed20. Epub 2013/01/01.
- Kinder BW, Sherman AC, Young LR, Hagaman JT, Oprescu N, Byrnes S, et al. Predictors for Clinical Trial Participation in the Rare Lung Disease Lymphangioleiomyomatosis. Respiratory Medicine. 2010;104(4):578-83.
- Kole A, Faurisson F. Rare diseases social epidemiology: analysis of inequalities. Advances in Experimental Medicine and Biology. 2010;686:223-50.
- Kruer MC, Steiner RD. The Role of Evidence-Based Medicine and Clinical Trials in Rare Genetic Disorders. Clinical Genetics. 2008;74(3):197-207.
- Lebioda A, Hulsebeck M, Plantor S. Orphan drugs in the german early benefit assessment- real world versus G-BA bureaucracy. Value in health. 2013; Conference: ISPOR 16th Annual European Congress Dublin Ireland.
- MacCoun R, Perlmutter S. Blind analysis: Hide results to seek the truth. Nature. 2015;526(7572):187-9. Epub 2015/10/10.
- Nony P, Kurbatova P, Bajard A, Malik S, Castellan C, Chabaud S, et al. A methodological framework for drug development in rare diseases. Orphanet Journal of Rare Diseases. 2014;9:164. Epub 2014/01/01.
- Panju AH, Bell CM. Policy alternatives for treatments for rare diseases. CMAJ : Canadian Medical Association Journal = Journal de l'Association Medicale canadienne. 2010;182(17):E787-92. Epub 2010/07/14.
- Parker S. The pooling of manpower and resources through the establishment of European reference networks and rare disease patient registries is a necessary area of collaboration for rare renal disorders. Nephrology, Dialysis, Transplantation: official publication of the European Dialysis and Transplant Association - European Renal Association. 2014;29 Suppl 4:iv9-14. Epub 2014/08/29.
- Potter BK, Khangura SD, Tingley K, Chakraborty P, Little J. Translating rare-disease therapies into improved care for patients and families: what are the right outcomes, designs, and engagement approaches in health-systems research? Genetics in Medicine: Official Journal of the American College of Medical Genetics. 2016;18(2):117-23. Epub 2015/04/10.
- Richesson RL, Sutphen R, Shereff D, Krischer JP. The Rare Diseases Clinical Research Network Contact Registry update: Features and functionality. Contemporary Clinical Trials. 2012;33(4):647-56.
- Shani S, Yahalom Z. Legal and regulatory aspects of orphan drugs. Pediatric Endocrinology Reviews. 2013;11 Suppl 1:110-5.
- Skoog M, Saarimäki JM, Gluud C, Sheinin M, Erlendsson K, Aamdal S. Transparency and registration in clinical research in the Nordic countries. Nordic Trial Alliance, NordForsk. 2015:1–108
- Suhr D. The open registry project as a tool for research and clinical trials. Molecular Genetics and Metabolism. 2012; Conference: 8th Annual Research Meeting of the Lysosomal Disease Network, WORLD Symposium 2012 San Diego, CA United States.
- Tamura RN, Krischer JP, Pagnoux C, Micheletti R, Grayson PC, Chen YF, et al. A small n sequential multiple assignment randomized trial design for use in rare disease research. Contemporary Clinical Trials. 2016;46:48-51. Epub 2015/11/21
- Teagarden JR, Unger TF, Hirsch G. Access and availability of orphan drugs in the United States: Advances or cruel hoaxes? Expert Opinion on Orphan Drugs. 2014;2(11):1147-50.
- The Academy of Medical Sciences; BBSRC; MRC Medical Research Council. Symposium Report. Reproducibility and reliability of biomedical research: improving research practice. Welcome Trust, 2015.
- Tudur Smith C, Williamson PR, Beresford MW. Methodology of clinical trials for rare diseases. Best Practice & Research in Clinical Rheumatology. 2014;28(2):247-62.
- Valdez R, Ouyang L, Bolen J. Public Health and Rare Diseases: Oxymoron No More. Preventing Chronic Disease. 2016;13:E05. Epub 2016/01/15.
